# Acquired Resistance of Lung Adenocarcinomas to Gefitinib or Erlotinib Is Associated with a Second Mutation in the EGFR Kinase Domain

**DOI:** 10.1371/journal.pmed.0020073

**Published:** 2005-02-22

**Authors:** William Pao, Vincent A Miller, Katerina A Politi, Gregory J Riely, Romel Somwar, Maureen F Zakowski, Mark G Kris, Harold Varmus

**Affiliations:** **1**Program in Cancer Biology and Genetics, Memorial Sloan-Kettering Cancer CenterNew York, New YorkUnited States of America; **2**Thoracic Oncology Service, Department of MedicineMemorial Sloan-Kettering Cancer Center, New York, New YorkUnited States of America; **3**Department of Pathology, Memorial Sloan-Kettering Cancer CenterNew York, New YorkUnited States of America; Genome Institute of SingaporeSingapore

## Abstract

**Background:**

Lung adenocarcinomas from patients who respond to the tyrosine kinase inhibitors gefitinib (Iressa) or erlotinib (Tarceva) usually harbor somatic gain-of-function mutations in exons encoding the kinase domain of the epidermal growth factor receptor (EGFR). Despite initial responses, patients eventually progress by unknown mechanisms of “acquired” resistance.

**Methods and Findings:**

We show that in two of five patients with acquired resistance to gefitinib or erlotinib, progressing tumors contain, in addition to a primary drug-sensitive mutation in *EGFR,* a secondary mutation in exon 20, which leads to substitution of methionine for threonine at position 790 (T790M) in the kinase domain. Tumor cells from a sixth patient with a drug-sensitive *EGFR* mutation whose tumor progressed on adjuvant gefitinib after complete resection also contained the T790M mutation. This mutation was not detected in untreated tumor samples. Moreover, no tumors with acquired resistance had *KRAS* mutations, which have been associated with primary resistance to these drugs. Biochemical analyses of transfected cells and growth inhibition studies with lung cancer cell lines demonstrate that the T790M mutation confers resistance to EGFR mutants usually sensitive to either gefitinib or erlotinib. Interestingly, a mutation analogous to T790M has been observed in other kinases with acquired resistance to another kinase inhibitor, imatinib (Gleevec).

**Conclusion:**

In patients with tumors bearing gefitinib- or erlotinib-sensitive *EGFR* mutations, resistant subclones containing an additional *EGFR* mutation emerge in the presence of drug. This observation should help guide the search for more effective therapy against a specific subset of lung cancers.

## Abstract 2

A specific secondary mutation in the kinase domain of the epidermal growth factor receptor can render cells insensitive to the two kinase inhibitors. This mutation was found in resistant tumors from three of six patients studied

## Introduction

Somatic gain-of-function mutations in exons encoding the epidermal growth factor receptor (EGFR) tyrosine kinase domain are found in about 10% of non-small cell lung cancers (NSCLCs) from the United States [1,2,3], with higher percentages observed in east Asia [2,4,5,6]. Some 90% of NSCLC-associated mutations occur as either multi-nucleotide in-frame deletions in exon 19, involving elimination of four amino acids, Leu-Arg-Glu-Ala, or as a single nucleotide substitution at nucleotide 2573 (T→G) in exon 21, resulting in substitution of arginine for leucine at position 858 (L858R). Both of these mutations are associated with sensitivity to the small-molecule kinase inhibitors gefitinib or erlotinib [1,2,3]. Unfortunately, nearly all patients who experience marked improvement on these drugs eventually develop progression of disease. While *KRAS* mutations have been associated with some cases of primary resistance to gefitinib or erlotinib [7], mechanisms underlying “acquired” or “secondary” resistance are unknown.

Acquired resistance to kinase-targeted anticancer therapy has been most extensively studied with imatinib, an inhibitor of the aberrant BCR-ABL kinase, in chronic myelogenous leukemia (CML). Mutations in the ABL kinase domain are found in 50%–90% of patients with secondary resistance to the drug (reviewed in [8]). Such mutations, which cluster in four distinct regions of the ABL kinase domain (the ATP binding loop, T315, M351, and the activation loop), interfere with binding of imatinib to ABL [9,10,11]. Crystallographic studies of various ABL mutants predict that most should remain sensitive to inhibitors that bind ABL with less stringent structural requirements. Using this insight, new small-molecule inhibitors have been identified that retain activity against the majority of imatinib-resistant BCR-ABL mutants [12,13].

Although imatinib inhibits different kinases in various diseases (BCR-ABL in CML, KIT or PDGFR-alpha in gastrointestinal stromal tumors [GISTs], and PDGFR-alpha in hypereosinophilic syndrome [HES]) (reviewed in [14]), some tumors that become refractory to treatment with imatinib appear to have analogous secondary mutations in the kinase-coding domain of the genes encoding these three enzymes. For example, in CML, a commonly found mutation is a C→T single nucleotide change that replaces threonine with isoleucine at position 315 (T315I) in the ABL kinase domain [9,10,11]. In GIST and HES, respectively, the analogous T670I mutation in KIT and T674I mutation in PDGFR-alpha have been associated with acquired resistance to this drug [15,16].

To determine whether lung cancers that acquire clinical resistance to either gefitinib or erlotinib display additional mutations in the EGFR kinase domain, we have examined the status of *EGFR* exons 18 to 24 in tumors from five patients who initially responded but subsequently progressed while on these drugs. These exons were also assessed in tumor cells from a sixth patient whose disease rapidly recurred while on gefitinib therapy after complete gross tumor resection. Because of the association of *KRAS* mutations with primary resistance to gefitinib and erlotinib [7], we also examined the status of *KRAS* in tumor cells from these six patients. In an effort to explain the selective advantage of cells with a newly identified “resistance” mutation in *EGFR—*a T790M amino acid substitution—we further characterized the drug sensitivity of putatively resistant EGFR mutants versus wild-type or drug-sensitive EGFR mutants, using both a NSCLC cell line fortuitously found to contain the T790M mutation and lysates from cells transiently transfected with wild-type and mutant *EGFR* cDNAs.

## Methods

### Tissue Procurement

Tumor specimens, including paraffin blocks, fine needle biopsies, and pleural effusions, were obtained through protocols approved by the Institutional Review Board of Memorial Sloan-Kettering Cancer Center (protocol 92–055 [7] and protocol 04–103 [Protocol S1]). All patients provided informed consent.

### Mutational Analyses of EGFR and KRAS in Lung Tumors

Genomic DNA was extracted from tumor specimens, and primers for *EGFR* (exons 18–24) and *KRAS2* (exon 2) analyses were as published [3,7]. All sequencing reactions were performed in both forward and reverse directions, and all mutations were confirmed at least twice from independent PCR isolates.

A specific exon 20 mutation (T790M) was also detected by length analysis of fluorescently labeled (FAM) PCR products on a capillary electrophoresis device (ABI 3100 Avant, Applied Biosystems, Foster City, California, United States), based on a new NlaIII restriction site created by the T790M mutation (2369 C→T), using the following primers: EGFR Ex20F, 5′-FAM- CTCCCTCCAGGAAGCCTACGTGAT-3′ and EGFR Ex20R 5′- TTTGCGATCTGCACACACCA-3′. Using serially mixed dilutions of DNA from NSCLC cell lines (H1975, L858R- and T790M-positive; H-2030, *EGFR* wild-type) for calibration, this assay detects the presence of the T790M mutation when H1975 DNA comprises 3% or more of the total DNA tested, compared to a sensitivity of 6% for direct sequencing (data not shown).

### RT-PCR

The following primers were used to generate *EGFR* cDNA fragments spanning exon 20: EGFR 2095F 5′- CCCAACCAAGCTCTCTTGAG-3′ and EGFR 2943R 5′- ATGACAAGGTAGCGCTGGGGG-3′. PCR products were ligated into plasmids using the TOPO TA-cloning kit (Invitrogen, Carlsbad, California, United States), as per manufacturer's instructions. Minipreps of DNA from individual clones were sequenced using the T7 priming site of the cloning vector.

### Functional Analyses of Mutant EGFRs

Two numbering systems are used for EGFR. The first denotes the initiating methionine in the signal sequence as amino acid −24. The second, used here, denotes the methionine as amino acid +1. Commercial suppliers of antibodies, such as the Y1068-specific anti-phospho-EGFR, use the first nomenclature. To be consistent, we consider Y1068 as Y1092. Likewise, the T790M mutation reported here has also been called T766M. Mutations were introduced into full-length wild-type and mutant *EGFR* cDNAs using a QuikChange Site-Directed Mutagenesis Kit (Stratagene, La Jolla, California, United States) and cloned into expression vectors as described [3]. The following primers were used to generate the deletion (del) L747–E749;A750P mutant: forward 5′- TAAAATTCCCGTCGCTATCAAGGAGCCAACATCTCCGAAAGCCAACAAGG-3′ and reverse 5′- CCTTGTTGGCTTTCGGAGATGTTGGCTCCTTGATAGCGACGGGAATTTTA-3′. The following primers were used to introduce the T790M mutation: forward 5′- AGCTCATCATGCAGCTCAT-3′ and reverse 5′- ATGAGCTGCATGATGAGCT-3′. The L858R mutant cDNA was generated previously [3]. All mutant clones were fully re-sequenced bidirectionally to ensure that no additional mutations were introduced. Various EGFRs were transiently expressed in 293T human embryonic kidney cells as published [3]. Cells were treated with different concentrations of gefitinib or erlotinib.

### Immunoblotting

See Methods and supplementary methods in [3] for details on cell lysis, immunoblotting, and antibody reagents. At least three independent experiments were performed for all analyses.

### Cell Culture

The NSCLC cell lines H1650, H1975, H2030, H2347, H2444, H358, and H1734 were purchased from American Type Culture Collection (Manassas, Virginia, United States). H3255 was a gift of B. Johnson and P. Janne. Cells were grown in complete growth medium (RPMI-1640; American Type Culture Collection catalog no. 30–2001) supplemented with 10% fetal calf serum, 10 units/ml penicillin, and 10 μg/ml streptomycin) at 37 °C and 5% CO_2_. For viability studies, cells were seeded in complete growth medium in black 96-well clear bottom ViewPlates (PerkinElmer, Wellesley, Massachusetts, United States) at a density of 5,000 (H1975 and H2030) or 7,500 cells per well (H3255). Following overnight incubation, cells were grown for 24 h in the supplemented RPMI-1640 medium with 0.1% serum. Cells (in supplemented RPMI-1640 medium containing 0.1% serum) were then incubated for 48 h in the continued presence of gefitinib or erlotinib.

### Viability Assay

Cell viability was assayed using Calcein AM (acetoxymethyl ester of Calcein, Molecular Probes, Eugene, Oregon, United States). Following incubation with gefitinib or erlotinib, monolayers were washed twice with PBS (containing calcium and magnesium) and incubated with 7.5 μmol Calcein AM in supplemented RPMI-1640 (no serum) for 30 min. Labeling medium was removed, and cells were washed three times with PBS. Calcein fluorescence (Ex, 485 nm; Em, 535 nM) was detected immediately using a Victor V multi-label plate reader (PerkinElmer). Three independent experiments were performed for each cell line; each experiment included four to eight replicates per condition.

## Results

### Case Reports

We identified secondary *EGFR* mutations in three of six individuals whose disease progressed on either gefitinib or erlotinib (Table 1). Brief case histories of these three patients are presented below.

**Table 1:**
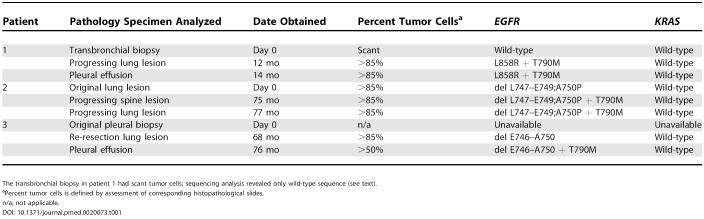
Specimens Analyzed in This Study for Mutations in the *EGFR* Tyrosine Kinase Domain (Exons 18 to 24) and *KRAS* (Exon 2)

The transbronchial biopsy in patient 1 had scant tumor cells; sequencing analysis revealed only wild-type sequence (see text)

^a^Percent tumor cells is defined by assessment of corresponding histopathological slides

n/a, not applicable

#### Patient 1

**Figure 1:**
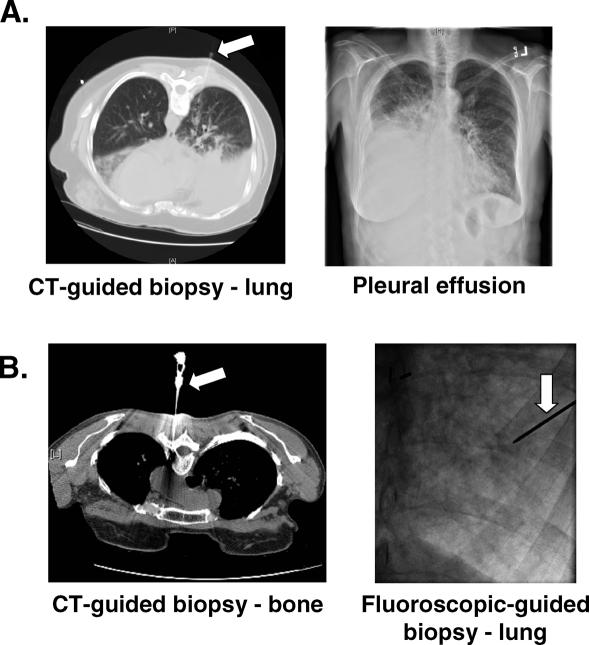
Re-Biopsy Studies (A.) Patient 1. CT-guided biopsy of progressing lung lesions after 10 mo on gefitinib (left panel). Two months later, fluid from a right-sided pleural effusion (right panel) was collected for molecular analysis. (B) Patient 2. CT-guided biopsy of a progressing thoracic spine lesion (left panel) and fluoroscopic-guided biopsy of a progressing lung lesion (right panel). The biopsy needles are indicated by white arrows.

This 63-y-old female “never smoker” (smoked less than 100 cigarettes in her lifetime) initially presented with bilateral diffuse chest opacities and a right-sided pleural effusion. Transbronchial biopsy revealed adenocarcinoma. Disease progressed on two cycles of systemic chemotherapy, after which gefitinib, 250 mg daily, was started. Comparison of chest radiographs obtained prior to starting gefitinib (Figure S1A, left panel) and 2 wk later (Figure S1A, middle panel) showed dramatic improvement. Nine months later, a chest radiograph revealed progression of disease (Figure S1A, right panel). Subsequently, the patient underwent a computed tomography (CT)–guided biopsy of an area in the right lung base (Figure 1A, left panel). Despite continued treatment with gefitinib, either with chemotherapy or at 500 mg daily, the pleural effusion recurred, 12 mo after initiating gefitinib (Figure 1A, right panel). Pleural fluid was obtained for molecular studies. In total, this patient had three tumor specimens available for analysis: the original lung tumor biopsy, a biopsy of the progressing lung lesion, and pleural fluid. However, re-review of the original transbronchial biopsy showed that it had scant tumor cells (Table 1).

#### Patient 2.

This 55-y-old woman with a nine pack-year history of smoking underwent two surgical resections within 2 y (right lower and left upper lobectomies) for bronchioloalveolar carcinoma with focal invasion. Two years later, her disease recurred with bilateral pulmonary nodules and further progressed on systemic chemotherapy. Thereafter, the patient began erlotinib, 150 mg daily. A baseline CT scan of the chest demonstrated innumerable bilateral nodules (Figure S1B, left panel), which were markedly reduced in number and size 4 mo after treatment (Figure S1B, middle panel). After 14 mo of therapy, the patient's dose of erlotinib was decreased to 100 mg daily owing to fatigue. At 23 mo of treatment with erlotinib, a CT scan demonstrated an enlarging sclerotic lesion in the thoracic spine. The patient underwent CT-guided biopsy of this lesion (Figure 1B, left panel), and the erlotinib dose was increased to 150 mg daily. After 25 mo of treatment, she progressed within the lung (Figure S1B, right panel). Erlotinib was discontinued, and a fluoroscopically guided core needle biopsy was performed at a site of progressive disease in the lung (Figure 1B, right panel). In total, this patient had three tumor specimens available for analysis: the original resected lung tumor, the biopsy of the enlarging spinal lesion, and the biopsy of the progressing lung lesion (Table 1).

#### Patient 3

This 55-y-old female “never smoker” was treated for nearly 4.5 y with weekly paclitaxel and trastuzumab [17] for adenocarcinoma with bronchioloalveolar carcinoma features involving her left lower lobe, pleura, and mediastinal lymph nodes. Treatment was discontinued owing to fatigue. Subsequently, the patient underwent surgical resection. Because of metastatic involvement of multiple mediastinal lymph nodes and clinical features known at that time to be predictive of response to gefitinib (female, never smoker, bronchioloalveolar variant histology), she was placed on “adjuvant” gefitinib 1 mo later (Figure S1C, left panel). This drug was discontinued after 3 mo when she developed a new left-sided malignant pleural effusion (Figure S1C, middle panel). Despite drainage and systemic chemotherapy, the pleural effusion recurred 4 mo later (Figure S1C, right panel), at which time pleural fluid was collected for analysis. In total, this patient had two clinical specimens available for analysis: tumor from the surgical resection and pleural fluid (Table 1).

### Patients' Tumors Contain *EGFR* Tyrosine Kinase Domain Mutations Associated with Sensitivity to EGFR Tyrosine Kinase Inhibitors

**Figure 2:**
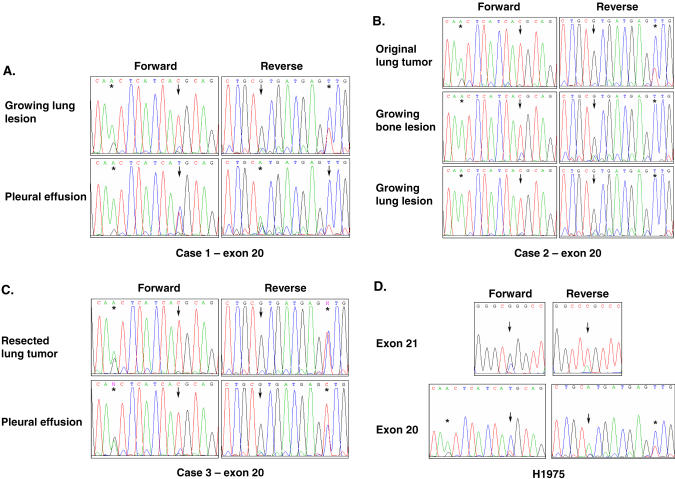
Sequencing Chromatograms with the T790M *EGFR* Exon 20 Mutation in Various Clinical Specimens and the NSCLC Cell Line H1975 (A–C) In all three patients—patient 1 (A), patient 2 (B), and patient 3 (C)—the secondary T790M mutation was observed only in lesions obtained after progression on either gefitinib or erlotinib. (D) Cell line H1975 contains both an exon 21 L858R mutation (upper panel) and the exon 20 T790M mutation (lower panel). The asterisks indicate a common SNP (A or G) at nucleotide 2361; the arrows indicate the mutation at nucleotide 2369 (C→T), which leads to substitution of methonine (ATG) for threonine (ACG) at position 790. In the forward direction, the mutant T peak is blue. In the reverse direction, the mutant peak is green, while the underlying blue peak represents an “echo” from the adjacent nucleotide.

We screened all available tumor samples from these three patients for previously described drug-sensitive *EGFR* mutations, by direct DNA sequencing of exons 19 and 21 [3]. Tumor samples from patient 1 showed a T→G change at nucleotide 2573, resulting in the exon 21 L858R amino acid substitution commonly observed in drug-responsive tumors. This mutation was present in the biopsy material from the progressing lung lesion (Figure S2A, upper panels) and from cells from the pleural effusion (Figure S2A, lower panels), both of which on cytopathologic examination consisted of a majority of tumor cells (Table 1). Interestingly, comparisons of the tracings suggest that an increase in copy number of the mutant allele may have occurred. Specifically, while the ratio of wild-type (nucleotide T) to mutant (nucleotide G) peaks at position 2573 was approximately 1:1 or 1:2 in the lung biopsy specimen (Figure S2A, upper panels), sequencing of DNA from the pleural fluid cells demonstrated a dominant mutant G peak (Figure S2A, lower panels). Consistent with this, a single nucleotide polymorphism (SNP) noted at nucleotide 2361 (A or G) demonstrated a corresponding change in the ratios of A:G, with a 1:1 ratio in the transbronchial biopsy, and a nearly 5:1 ratio in the pleural fluid (Figure 2A). Notably, we did not detect the 2573 T→G mutation in the original transbronchial biopsy specimen (Table 1; data not shown). As stated above, this latter specimen contained scant tumor cells, most likely fewer than needed for detection of an *EGFR* mutation by direct sequencing (see [7]).

All three specimens from patient 2, including the original lung tumor and the two metastatic samples from bone and lung, showed an exon 19 deletion involving elimination of 11 nucleotides (2238–2248) and insertion of two nucleotides, G and C (Figure S2B, all panels; Table 1). These nucleotide changes delete amino acids L747–E749 and change amino acid 750 from alanine to proline (A750P). A del L747–E749;A750P mutation was previously reported with different nucleotide changes [2]. In all samples from patient 2, the wild-type sequence predominated at a ratio of about 3:1 over the mutant sequence.

Both of the available tumor samples from patient 3 contained a deletion of 15 nucleotides (2236–2250) in exon 19 (Table 1; data not shown), resulting in elimination of five amino acids (del E746–A750). This specific deletion has been previously reported [3]. The ratio of mutant to wild-type peaks was approximately 1:1 in both specimens (data not shown).

Collectively, these results demonstrate that tumors from all three patients contain *EGFR* mutations associated with sensitivity to the tyrosine kinase inhibitors gefitinib and erlotinib. In addition, these data show that within individual patients, metastatic or recurrent lesions to the spine, lung, and pleural fluid contain the same mutations. These latter observations support the idea that relapsing and metastatic tumor cells within individuals are derived from original progenitor clones.

### A Secondary Missense Mutation in the *EGFR* Kinase Domain Detected in Lesions That Progressed while on Treatment with Either Gefitinib or Erlotinib

To determine whether additional mutations in the *EGFR* kinase domain were associated with progression of disease in these patients, we performed direct sequencing of all of the exons (18 through 24) encoding the EGFR catalytic region in the available tumor specimens.

Analysis of patient 1's pre-gefitinib specimen, which contained scant tumor cells (Table 1; see above), not surprisingly showed only wild-type *EGFR* sequence (Table 1; data not shown). However, careful analysis of the exon 20 sequence chromatograms in both forward and reverse directions from this patient's lung biopsy specimen obtained after disease progression on gefitinib demonstrated an additional small peak at nucleotide 2369, suggesting a C→T mutation (Figure 2A, upper panels; Table 1). This nucleotide change leads to substitution of methionine for threonine at position 790 (T790M). The 2369 C→T mutant peak was even more prominent in cells from the patient's pleural fluid, which were obtained after further disease progression on gefitinib (Figure 2A, lower panels; Table 1). The increase in the ratio of mutant to wild-type peaks obtained from analyses of the lung specimen and pleural fluid paralleled the increase in the ratio of the mutant G peak (leading to the L858R mutation) to the wild-type T peak at nucleotide 2573 (see above; Figure S2A), as well as the increase in the ratio of the A:G SNP at position 2361 (Figure 2A). Collectively, these findings imply that the exon 20 T790M mutation was present on the same allele as the exon 21 L858R mutation, and that a subclone of cells harboring these mutations emerged during drug treatment.

In patient 2, the tumor-rich sample obtained prior to treatment with erlotinib did not contain any additional mutations in the exons encoding the *EGFR* tyrosine kinase domain (Figure 2B, upper panels; Table 1). By contrast, her progressing bone and lung lesions contained an additional small peak at nucleotide 2369, suggesting the existence of a subclone of tumor cells with the same C→T mutation observed in patient 1 (Figure 2B, middle and lower panels; Table 1). The relative sizes of the 2369 T mutant peaks seen in these latter two samples appeared to correlate with the relative size of the corresponding peaks of the exon 19 deletion (Figure S2B). Interestingly, the SNP at nucleotide 2361 (A or G) was detected in specimens from patient 2 before but not after treatment with erlotinib, suggesting that one *EGFR* allele underwent amplification or deletion during the course of treatment (Figure S2B).

Patient 3 showed results analogous to those of patient 2. A tumor-rich pre-treatment specimen did not demonstrate *EGFR* mutations other than the del E746–A750 exon 19 deletion; specifically, in exon 20, no secondary changes were detected (Figure 2C, upper panels; Table 1). However, analysis of DNA from cells in the pleural effusion that developed after treatment with gefitinib showed the C→T mutation at nucleotide 2369 in exon 20 (Figure 2C, lower panels; Table 1), corresponding to the T790M mutation described above. There was no dramatic change between the two samples in the ratio of the A:G SNP at position 2361. The mutant 2369 T peak was small, possibly because gefitinib had been discontinued in this patient for 4 mo at the time pleural fluid tumor cells were collected; thus, there was no selective advantage conferred upon cells bearing the T790M mutation.

To determine whether the 2369 C→T mutation was a previously overlooked *EGFR* mutation found in NSCLCs, we re-reviewed exon 20 sequence tracings derived from analysis of 96 fresh-frozen resected tumors [3] and 59 paraffin-embedded tumors [7], all of which were removed from patients prior to treatment with an EGFR tyrosine kinase inhibitor. We did not detect any evidence of the T790M mutation in these 155 tumors (data not shown; see Discussion). Collectively, our results suggest that the T790M mutation is associated with lesions that progress while on gefitinib or erlotinib. Moreover, at least in patients 1 and 2, the subclones of tumor cells bearing this mutation probably emerged between the time of initial treatment with a tyrosine kinase inhibitor and the appearance of drug resistance.

In three additional patients (case histories not described here) with lung adenocarcinomas who improved but subsequently progressed on therapy with either gefitinib or erlotinib, we examined DNA from tumor specimens obtained during disease progression. In all three patients, we found *EGFR* mutations associated with drug sensitivity (all exon 19 deletions). However, we did not find any additional mutations in exons 18 to 24 of *EGFR,* including the C→T change at position 2369 (data not shown). These results imply that alternative mechanisms of acquired drug resistance exist.

### Patients' Progressive Tumors Lack *KRAS* Mutations

Mutations in exon 2 of *KRAS2* occur in about one-fourth of NSCLCs. Such mutations rarely, if ever, accompany *EGFR* mutations and are associated with primary resistance to gefitinib or erlotinib [7]. To evaluate the possibility that secondary *KRAS* mutations confer acquired resistance to these drugs, we performed mutational profiling of *KRAS2* exon 2 from tumor specimens from patients 1 to 3, as well as the three additional patients lacking evidence of the T790M mutation. None of the specimens contained any changes in *KRAS* (Table 1; data not shown), indicating that *KRAS* mutations were not responsible for drug resistance and tumor progression in these six patients.

### An Established NSCLC Cell Line Also Contains Both T790M and L858R Mutations

We profiled the *EGFR* tyrosine kinase domain (exons 18 to 24) and *KRAS* exon 2 in eight established NSCLC lines (Table 2). Surprisingly, one cell line—H1975—contained the same C→T mutation at position 2369 (T790M) as described above (Figure 2D, lower panel). This cell line had previously been shown by others to contain a 2573 T→G mutation in exon 21 (L858R) [18], which we confirmed (Figure 2D, upper panel); in addition, H1975 was reported to be more sensitive to gefitinib inhibition than other lung cancer cell lines bearing wild-type *EGFR* [18]. Only exons 19 and 21 were apparently examined in this published study.

**Table 2:**
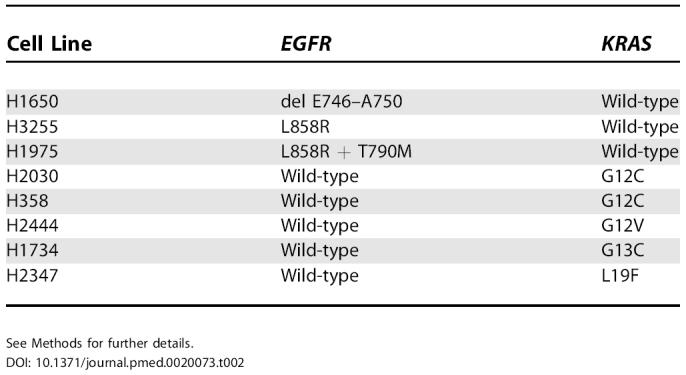
Status of NSCLC Cell Lines Analyzed for *EGFR* Tyrosine Kinase Domain (Exons 18 to 24) and *KRAS* (Exon 2) Mutations

See Methods for further details

In our own analysis of H1975 (exons 18 to 24), the mutant 2369 T peak resulting in the T790M amino acid substitution was dominant, suggesting an increase in copy number of the mutant allele in comparison to the wild-type allele. The ratio of mutant to wild-type peaks was similar to that of the mutant 2573 G (corresponding to the L858R amino acid substitution) to wild-type T peaks (Figure 2D, all panels), implying that the T790M and L858R mutations were in the same amplified allele. To further investigate this possibility, we performed RT-PCR to generate cDNAs that spanned exon 20 of *EGFR* and included sequences from exon 19 and 21. PCR products were then cloned, and individual colonies were analyzed for *EGFR* mutations. Sequencing chromatograms of DNA from four of four clones showed both the 2369 C→T and 2573 T→G mutations, confirming that both mutations were in the same allele (data not shown).

Other NSCLC cell lines carried either *EGFR* or *KRAS* mutations, but none had both (Table 2). As reported, H3255 contained an L858R mutation [19] and H1650 contained an exon 19 deletion [18]. No other cell lines analyzed contained additional mutations in the exons encoding the EGFR tyrosine kinase domain.

### A Novel PCR Restriction Fragment Length Polymorphism Assay Independently Confirms the Absence or Presence of the T790M Mutation

**Figure 3:**
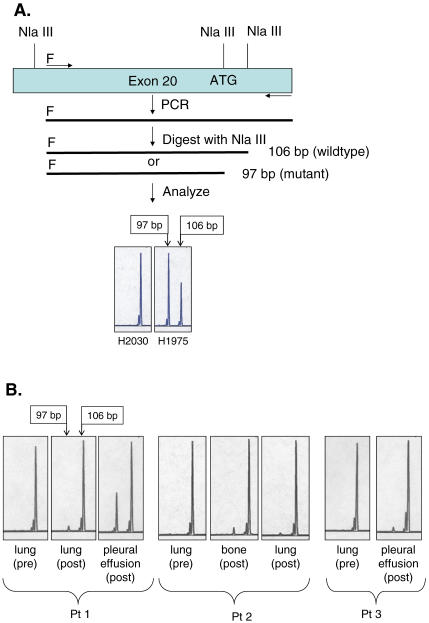
A Novel PCR-RFLP Assay Independently Confirms Presence of the T790M Mutation in Exon 20 of the *EGFR* Kinase Domain (A) Design of the assay (see text for details). “F” designates the fluorescent label, FAM. At the bottom of this panel, the assay demonstrates with the 97-bp NlaIII cleavage product the presence of the T790M mutation in the H1975 cell line; this product is absent in H2030 DNA. The 106-bp NlaIII cleavage product is generated by digestion of wild-type *EGFR*. (B) The PCR-RFLP assay demonstrates that pre-drug tumor samples from the three patients lack detectable levels of the mutant 97-bp product, while specimens obtained after disease progression contain the T790M mutation. Pt, patient.

As stated above, the mutant peaks suggestive of a T790M mutation in exon 20 were small in some sequence chromatograms. To eliminate the possibility that these peaks were due to background “noise,” we sought to confirm the presence of the 2369 C→T mutation in specific samples, by developing an independent test, based on a fluorescence detection assay that takes advantage of a PCR restriction fragment length polymorphism (PCR-RFLP) generated by the specific missense mutation. After PCR amplification with exon-20-specific primers spanning nucleotide 2369, wild-type sequence contains specific NlaIII sites, which upon digestion yield a 106-bp product (see Methods; Figure 3A). Presence of the mutant 2369 T nucleotide creates a new NlaIII restriction digest site, yielding a slightly shorter product (97 bp), readily detected by fluorescent capillary electrophoresis. This test is about 2 -fold more sensitive than direct sequencing (see Methods; data not shown).

We first used DNA from the H1975 cell line (which contains both T790M and L858R mutations) to confirm the specificity of the PCR-RFLP assay. As expected, analysis of these cells produced both the 97- and 106-bp fragments. By contrast, analysis of DNA from H2030 (which contains wild-type *EGFR;* Table 2) showed only the 106-bp fragment (Figure 3A). These data show that this test can readily indicate the absence or presence of the mutant allele in DNA samples. However, this test was only semi-quantitative, as the ratio of the mutant 97-bp product versus the wild-type 106-bp product varied in independent experiments from approximately 1:1 to 2:1.

We next used this PCR-RFLP assay to assess various patient samples for the presence of the specific 2369 C→T mutation corresponding to the T790M amino acid substitution. DNA from the progressing bone and lung lesions in patient 1 produced both the 97- and 106-bp fragments, but DNA from the original lung tumor did not (Figure 3B). The ratio of mutant to wild-type products was higher in the cells from the pleural fluid, consistent with the higher peaks seen on the chromatograms from direct sequencing of exon 20 (see Figure 2A). Likewise, DNA from progressive lesions from patients 2 and 3 yielded both 97- and 106-bp fragments in the PCR-RFLP assay (Figure 3B), whereas the pre-treatment specimens did not produce the 97-bp product. Collectively, these data from an independent assay confirm that the T790M mutation was present in progressing lesions from all three patients. We were also unable to detect the T790M mutation in any specimens from the three additional patients with acquired resistance that failed to demonstrate secondary mutations in *EGFR* exons 18 to 24 by direct sequencing (data not shown).

### Biochemical Properties of EGFR Mutants

To determine how the T790M mutation would affect EGFR proteins already containing mutations associated with sensitivity to EGFR tyrosine kinase inhibitors, we introduced the specific mutation into *EGFR* cDNAs that encoded the exon 21 and 19 mutations found in patients 1 and 2, respectively. Corresponding proteins ([i] L858R and L858R plus T790M, [ii] del L747–E749;A750P and del L747–E749;A750P plus T790M, and [iii] wild-type EGFR and wild-type EGFR plus T790M) were then produced by transient transfection with expression vectors in 293T cells, which have very low levels of endogenous EGFR [3]. Various lysates from cells that were serum-starved and pre-treated with gefitinib or erlotinib were analyzed by immunoblotting. Amounts of total EGFR (t-EGFR) were determined using an anti-EGFR monoclonal antibody, and actin served as an indicator of relative levels of protein per sample. To assess the drug sensitivity of the various EGFR kinases in surrogate assays, we used a Y1092-phosphate-specific antibody (i.e., phospho-EGFR [p-EGFR]) to measure the levels of “autophosphorylated” Tyr-1092 on EGFR in relation to levels of t-EGFR protein. We also assessed the global pattern and levels of induced tyrosine phosphorylation of cell proteins by using a generalized anti-phosphotyrosine reagent (RC-20).

**Figure 4:**
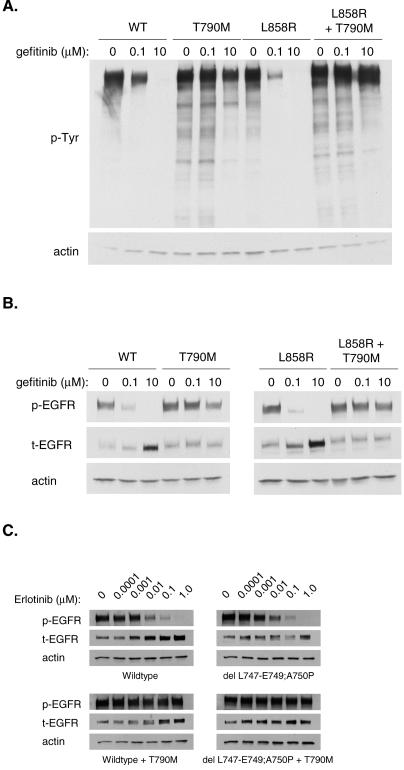
EGFR Mutants Containing the T790M Mutation Are Resistant to Inhibition by Gefitinib or Erlotinib 293T cells were transiently transfected with plasmids encoding wild-type (WT) EGFR or EGFR mutants with the following changes: T790M, L858R, L858R + T790M, del L747–E749;A750P, or del L747–E749;A750P + T790M. After 36 h, cells were serum-starved for 24 h, treated with gefitinib or erlotinib for 1 h, and then harvested for immunoblot analysis using anti-p-EGFR (Y1092), anti-t-EGFR, anti-phosphotyrosine (p-Tyr), and anti-actin antibodies as described in Methods. The EGFR T790M mutation, in conjunction with either wild-type EGFR or the drug-sensitive L858R EGFR mutant, prevents inhibition of tyrosine phosphorylation (A) or p-EGFR (B) by gefitinib. Analogously, the T790M mutation, in conjunction with the drug-responsive del L747–E749;A750P EGFR mutant, prevents inhibition of p-EGFR by erlotinib (C).

Gefitinib inhibited the activity of wild-type and L858R EGFRs progressively with increasing concentrations of drug, as demonstrated by a reduction of tyrosine-phosphorylated proteins (Figure 4A) and a decrease in p-EGFR:t-EGFR ratios (Figure 4B). By contrast, wild-type and mutant EGFRs containing the T790M mutation did not display a significant change in either phosphotyrosine induction or p-EGFR:t-EGFR ratios (Figure 4A and 4B). Similar results were obtained using erlotinib against wild-type and del E747–L747;A750P EGFRs in comparison to the corresponding mutants containing the T790M mutation (Figure 4C). These results suggest that the T790M mutation may impair the ability of gefitinib or erlotinib to inhibit EGFR tyrosine kinase activity, even in EGFR mutants (i.e., L858R or an exon 19 deletion) that are clinically associated with drug sensitivity.

### Resistance of a NSCLC Cell Line Harboring Both T790M and L858R Mutations to Gefitinib or Erlotinib

**Figure 5:**
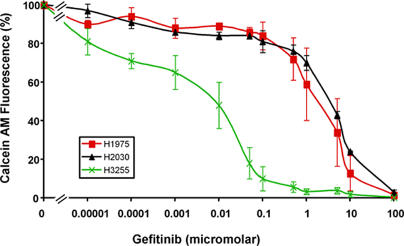
Sensitivity to Gefitinib Differs Among NSCLC Cell Lines Containing Various Mutations in *EGFR* or *KRAS* The three indicated NSCLC cell lines, H3255 (L858R mutation), H1975 (both T790M and L858R mutations), and H2030 (wild-type *EGFR,* mutant *KRAS*) (see Table 2), were grown in increasing concentrations of gefitinib, and the density of live cells after 48 h of treatment was measured using a Calcein AM fluorescence assay. Fluorescence in vehicle-treated cells is expressed as 100%. Results are the mean ± standard error of three independent experiments in which there were four to eight replicates of each condition. Similar results were obtained with erlotinib (see Figure S3).

To further explore the functional consequences of the T790M mutation, we determined the sensitivity of various NSCLC cells lines grown in the presence of either gefitinib or erlotinib, using an assay based upon Calcein AM. Uptake and retention of this fluorogenic esterase substrate by vehicle- versus drug-treated live cells allows for a comparison of relative cell viability among cell lines [20]. The H3255 cell line, which harbors the L858R mutation and no other *EGFR* TK domain mutations (Table 2), was sensitive to treatment with gefitinib, with an IC_50_ of about 0.01 μmol (Figure 5). By contrast, the H1975 cell line, which contains both L858R and T790M mutations (Table 2), was approximately 100-fold less sensitive to drug, with an IC_50_ of about 1 μmol (Figure 5). In fact, the sensitivity of H1975 cells was more similar to that of H2030, which contains wild-type *EGFR* (exons 18 to 24) and mutant *KRAS* (Figure 5). Very similar results were obtained with erlotinib (Figure S3).

## Discussion

Specific mutations in the tyrosine kinase domain of *EGFR* are associated with sensitivity to either gefitinib or erlotinib, but mechanisms of acquired resistance have not yet been reported. Based upon analogous studies in other diseases with another kinase inhibitor, imatinib, a single amino acid substitution from threonine to methionine at position 790 in the wild-type EGFR kinase domain was predicted to lead to drug resistance, even before the association of exon 19 and 21 mutations of *EGFR* with drug responsiveness in NSCLC was reported. The T790M mutation was shown in vitro in the context of wild-type EGFR to confer resistance to gefitinib [21] and a related quinazoline inhibitor, PD153035 [22].

We show here, through molecular analysis of tumor material from three patients and one NSCLC cell line, as well as additional biochemical studies, that acquired clinical drug resistance to gefitinib or erlotinib is indeed associated with the T790M mutation. Importantly, we find that the T790M mutation confers drug resistance not just to wild-type EGFR but also to mutant EGFRs associated with clinical responsiveness to EGFR tyrosine kinase inhibitors [1,2,3]. Our results further demonstrate that an analogous mechanism of acquired resistance exists for imatinib and EGFR tyrosine kinase inhibitors (Table 3), despite the fact that the various agents target different kinases in distinct diseases.

**Table 3:**
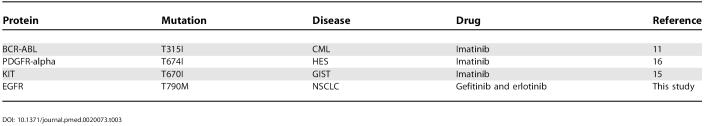
Analogous Mutations in Four Kinases Associated with Resistance to Kinase Inhibitors

In tumors from patients not treated with either gefitinib or erlotinib, the 2369 C→T mutation (T790M) appears to be extremely rare. We have not identified this mutation in 155 tumors (see above), and among nearly 1,300 lung cancers in which analysis of *EGFR* exons 18 to 21 has been performed [1,2,3,4,5,6], only one tumor (which also harbored an L858R mutation) was reported to contain the T790M mutation. Whether the patient from which this tumor was resected had received gefitinib or erlotinib is unclear, and the report did not note an association with acquired resistance to either drug [5].

How tumor cells bearing the T790M mutation emerge within gefitinib- or erlotinib-treated patients is a matter of investigation. Subclones bearing this mutation could arise de novo during treatment*.* However, based upon analogous studies in CML, it is also possible that NSCLC subclones bearing this secondary mutation pre-exist within the primary tumor clone in individual patients, albeit at low frequency [23]. In either scenario, treatment with gefitinib or erlotinib subsequently allows these resistant subclones to become apparent, because most cells bearing sensitivity-conferring mutations die, while cells with the T790M mutation persist.

From analysis of the crystal structure of the EGFR kinase domain bound to erlotinib, it is has been shown that the wild-type threonine residue at position 790 is located in the hydrophobic ATP-binding pocket of the catalytic region, where it forms a critical hydrogen bond with the drug [24]. The related compound, gefitinib, is predicted to interact with this threonine residue as well. Substitution of the threonine at position 790 by a larger residue like methionine would probably result in steric clash with the aromatic moieties on these two drugs [25]. By contrast, ATP would likely not depend on the accessibility of the same hydrophobic cavity and is therefore probably not affected by the incorporation of a bulky methionine side chain [25]. Consistent with this, the T790M mutation has been shown not to abrogate the catalytic activity of wild-type EGFR [22].

The T790M mutation could also affect the kinase activity or alter the substrate specificity of mutant EGFRs, such that a proliferative advantage would be conferred upon cells bearing the mutation. Consistent with this, the H1975 NSCLC cell line reported here to contain both T790M and L858R did not to our knowledge undergo any prior treatment with gefitinib or erlotinib; the doubly mutated cells must have become dominant over time through multiple passages in vitro. This scenario could explain the seemingly contradictory report by others who found the H1975 cell line to be highly sensitive to gefitinib [18]; our H1975 cells could represent a subclone that emerged over time. Analysis of earlier passages of H1975 cells for the T790M mutation would be informative in this regard.

Recently, new small-molecule inhibitors have been identified that retain activity against the majority of imatinib-resistant BCR-ABL mutants. The new drugs bind to ABL in an “open” conformation, as opposed to imatinib, which binds ABL in a “closed” conformation [12,13]. Analogously, it may be possible to find EGFR tyrosine kinase inhibitors that bind to the EGFR kinase domain in different ways than gefitinib and erlotinib. For example, the crystal structure of another EGFR inhibitor, lapatinib (GW572016), was recently solved bound to EGFR [26]. This study revealed that the quinazoline rings of erlotinib and lapatinib interact differently with the EGFR kinase domain, suggesting that while the T790M mutation may affect inhibition by erlotinib and gefitinib, it may not affect inhibition of EGFR by compounds similar to lapatinib. To our knowledge, no NSCLC patient who initially responded to but then progressed on either gefitinib or erlotinib has yet been treated with lapatinib.

In some of the patient specimens analyzed, the actual sequencing peaks demonstrating the T790M mutation were smaller than originally anticipated. These results differ from those of acquired resistance mutation in CML [10], GIST [15,27], and HES [16]. However, in contrast to all of these diseases, in which tumor cells are readily accessible, lung-cancer-related tumors are more difficult to access, as illustrated by the limited manner in which we were able to obtain tumor cells from various sites of disease (see Figure 1). Moreover, re-biopsy of patients with lung cancer is not routinely performed. The use of position emission tomography scans to identify the most metabolically active lesions for biopsy could possibly circumvent this factor in the future, as long as such lesions are resectable. Additionally, as more molecularly tailored treatment options become available for lung cancer, re-biopsy of progressive sites of disease should become a standard procedure, especially for patients on clinical trials of targeted agents.

Since tumor specimens from three additional patients with acquired resistance to EGFR tyrosine kinase inhibitors did not demonstrate the T790M mutation, this specific lesion does not account for all mechanisms of acquired resistance to gefitinib or erlotinib. Given the paradigm established with imatinib, other drug-resistance mutations in *EGFR,* either within or outside the tyrosine kinase domain, are likely to exist. It is also possible that *EGFR* amplification itself plays a role in acquired resistance, since imatinib-resistant clones have been shown to lack resistance mutations but contain amplified copies of *BCR-ABL* [11,28]. Nonetheless, studies presented here provide a basis for the rational development of “second generation” kinase inhibitors for use in NSCLC.

## Supporting Information

Figure S1 Imaging Studies from Patients 1, 2, and 3 (A) Patient 1. Serial chest radiographs from before (day 0) and during gefitinib treatment (14 d and 9 mo), demonstrating initial response and subsequent progression.
(B) Patient 2. Serial CT studies of the chest before (day 0) and during erlotinib treatment (4 mo and 25 mo), demonstrating initial response and subsequent progression.
(C) Patient 3. Serial chest radiographs before (day 0) and during adjuvant gefitinib treatment (3 mo), following complete resection of grossly visible disease. The left-sided pleural effusion seen at 3 mo recurred 4 mo later, at which time fluid was collected for molecular analysis.
(951 KB PPT).

Figure S2 Sequencing Chromatograms with the *EGFR* Exon 19 and 21 Mutations Identified in Patients 1 and 2 (A) Status of *EGFR* exon 21 in tumor specimens from patient 1. DNA from the growing lung lesion and the pleural effusion demonstrated a heterozygous T→G mutation at position 2573, leading to the common L858R amino acid substitution.
(B) All three specimens from patient 2 showed the same heterozygous exon 19 deletion, removing residues 747–749 and changing the alanine at position 750 to proline.
(104 KB PPT).

Figure S3 Sensitivity to Erlotinib Differs among NSCLC Cell Lines Containing Various Mutations in *EGFR* or *KRAS*
See legend for Figure 5.
(153 KB PPT).

Protocol S1 Memorial Sloan-Kettering Cancer Center IRB Protocol 04–103 (566 KB PDF).

### Accession Numbers

The LocusLink (http://www.ncbi.nlm.nih.gov/LocusLink/) accession number for the *KRAS2* sequence discussed in this paper is 3845; the GenBank (http://www.ncbi.nlm.nih.gov/Genbank/) accession number for the *KRAS2* sequence discussed in this paper is NT_009714.16. Reference *EGFR* sequence was obtained from LocusLink accession number 1956 and GenBank accession number NT_033968.

**Patient Summary**

**Background**

Normal cells in our body have safety mechanisms that keep them from growing out of control. Tumor cells have somehow found ways around these safety mechanisms, in some cases through activating particular growth-promoting genes. One of these, the EGFR gene, is often activated in lung cancer. Two drugs, gefitinib (also known as Iressa) and erlotinib (also called Tarceva), have been developed to inhibit activated EGFR, and studies have shown that they can shrink tumors in some patients. Most patients who respond to these drugs have tumors that carry an alteration (or mutation) in the EGFR gene, which somehow makes their tumors responsive to the drugs.

**Why Was This Study Done?**

In those patients in whom the drugs work, the tumors shrink initially, but after a while they stop responding and the cancer comes back. The cancer has, as researchers describe it, become resistant to the drugs. Understanding how tumors become resistant is important to develop new and better drugs.

**What Did the Researchers Do?**

They asked patients who initially responded to erlotinib or gefitinib but then became resistant to consent to studies allowing further analysis of tumor tissue during and after drug treatment. They then re-examined the EGFR gene in these tumor samples.

**What Did They Find?**

They found that tumors from all patients carried mutations in the EGFR gene that are known to make them responsive to the drugs. In addition, three of the post-treatment tumors had an identical second mutation in their EGFR gene. Biochemical studies showed that these secondary alterations made the original drug-sensitive EGFR less sensitive to drug treatment. The numbers are small but suggest that this secondary resistance mutation could be quite common. Tumor cells from the three other patients didn't have this mutation, which suggests that there are other ways for lung cancers to become resistant to gefitinib and erlotinib.

**What Next?**

Larger studies are needed to confirm that this particular mutation is a major cause of resistance against the two drugs. It is also important to find out what causes resistance in the other cases. And knowing about this resistance mutation will help researchers to develop drugs that will work even against tumors with the mutation.

**More Information Online**

The following pages contain some information on the EGFR kinase inhibitors.

U. S. Food and Drug Administration information page on Iressa (gefitinib): http://www.fda.gov/cder/drug/infopage/iressa/iressaQ&A.htm

Cancer Research UK information page about erlotinib (Tarceva): http://www.cancerhelp.org.uk/help/default.asp?page=10296

## Abbreviations

CML: chronic myelogenous leukemia
CT: computed tomography
del: deletion
EGFR: epidermal growth factor receptor
GIST: gastrointestinal stromal tumor
HES: hypereosinophilic syndrome
NSCLC: non-small cell lung cancer
p-EGFR: phospho-EGFR
PCR-RFLP: PCR restriction fragment length polymorphism
SNP: single nucleotide polymorphism
t-EGFR: total EGFR
