# Supplementary material for: Acquired Resistance of Lung Adenocarcinomas to Gefitinib or Erlotinib Is Associated with a Second Mutation in the EGFR Kinase Domain
Source: PLoS Med. 2005 Feb 22;2(3):e73. doi: 10.1371/journal.pmed.0020073 (PMC549606; doi:10.1371/journal.pmed.0020073)
Supplement: Supplementary file 1 [file pmed.0020073.sd001.pdf]

Memorial Sloan-Kettering Cancer Center
IRB Protocol

IRB#: 04-103A(1)

Molecular Determinants of Acquired Clinical Resistance to Erlotinib and Gefitinib in Non-Small-Cell Lung Cancer

THERAPEUTIC/DIAGNOSTIC PROTOCOL

Principal Investigator: Vincent A. Miller

Co-Principal Investigator(s): Mark G. Kris, William Pao

Investigator(s):

Thoracic Oncology: Naiyer Rizvi, Lee M. Krug, Jorge E. Gomez, Herbert F. Oettgen, Christopher G. Azzoli, Gregory J. Riely, Megan Dunne, Leslie Tyson, Barbara Pizzo, Amy Farmer

Thoracic Surgery: Valerie Rusch, Manjit Bains, Robert Downey, Raja Flores, Bernard Park

Pathology: Maureen Zakowski, Mark Ladanyi

Biostatistics: Ennapadam Venkatraman

Interventional Radiology: Anne Covey

Radiology: Robert T. Heelan

Consenting Professional(s): Mark G. Kris, Naiyer Rizvi, Lee M. Krug, Jorge E. Gomez, Herbert F. Oettgen, Christopher G. Azzoli, William Pao, Gregory J. Riely, Valerie Rusch, Manjit Bains, Robert Downey, Raja Flores, Bernard Park

Please Note: A Consenting Professional must have completed the mandatory Human Subjects Education and Certification Program.

Memorial Sloan-Kettering Cancer Center
1275 York Ave.
New York, NY 10021

IRB PB

Amended: 12/14/04

Table of Contents

THERAPEUTIC/DIAGNOSTIC PROTOCOL 1
1.0 PROTOCOL SUMMARY AND/OR SCHEMA 1
2.0 OBJECTIVES AND SCIENTIFIC AIMS 1
3.0 BACKGROUND AND RATIONALE 1
4.0 OVERVIEW OF STUDY DESIGN/INTERVENTION 4
4.1 DESIGN 4
4.2 INTERVENTION 4
5.0 THERAPEUTIC/DIAGNOSTIC AGENTS 5
6.0 CRITERIA FOR SUBJECT ELIGIBILITY 5
6.1 SUBJECT INCLUSION CRITERIA 5
7.0 RECRUITMENT PLAN 5
8.0 PRETREATMENT EVALUATION 6
9.0 TREATMENT/INTERVENTION PLAN 6
10.0 EVALUATION DURING TREATMENT/INTERVENTION 6
11.0 TOXICITIES/SIDE EFFECTS 8
12.0 CRITERIA FOR THERAPEUTIC RESPONSE/OUTCOME ASSESSMENT 8
13.0 CRITERIA FOR REMOVAL FROM STUDY 8
14.0 BIOSTATISTICS 8
15.0 SUBJECT REGISTRATION AND RANDOMIZATION PROCEDURES 9
15.1 SUBJECT REGISTRATION 9
15.2 RANDOMIZATION 10
16.0 DATA MANAGEMENT ISSUES 10
16.1 QUALITY ASSURANCE 10
16.2 DATA AND SAFETY MONITORING 10
17.0 PROTECTION OF HUMAN SUBJECTS 11
17.1 PRIVACY 12
17.2 SERIOUS ADVERSE EVENT (SAE) REPORTING 12
18.0 INFORMED CONSENT PROCEDURES 12
18.1 RESEARCH AUTHORIZATION 13
19.0 REFERENCE(S) 14
20.0 APPENDICES 14

1.0 PROTOCOL SUMMARY AND/OR SCHEMA

The goal of this protocol is to determine mechanisms of resistance to epidermal growth factor (EGFR) tyrosine kinase inhibitors (EGFR-TKI) in non-small cell lung cancer (NSCLC). A number of trials have shown the EGFR-TKIs erlotinib and gefitinib to be active agents in the treatment of NSCLC [1]. Clinically these drugs have been noted to produce dramatic but infrequent responses. Recently, mutations in the epidermal growth factor receptor have been shown to correlate with sensitivity to gefitinib [2,3]. However, we know that most patients who have initial responses to EGFR-TKI eventually progress. The mechanism of acquired clinical resistance to these inhibitors in patients is unknown.

This is a protocol to obtain by repeat biopsy tissue of patients with non-small cell lung cancer who have had previous clinical response to erlotinib or gefitinib and subsequently experience progression of disease. The tissues and other specimens will be used to carry out laboratory studies to explore the molecular basis of sensitivity and resistance to erlotinib and gefitinib.

2.0 OBJECTIVES AND SCIENTIFIC AIMS

Primary objective:

- To compare EGFR gene sequence in patients upon relapse with EGFR gene sequence prior to treatment with gefitinib or erlotinib.

Secondary objective:

- To compare EGFR gene copy number in patients who have progressed after initially responding to either erlotinib or gefitinib with those preceding treatment.
- To identify and determine the frequency of mutations in the tyrosine kinase domain of *EGFR* in patients with acquired resistance to erlotinib and gefitinib.

3.0 BACKGROUND AND RATIONALE

Non-small cell lung cancer (NSCLC) is the leading cause of cancer death in the United States with over 160,000 people dying each year [4]. The median survival of patients with advanced NSCLC is less than one year.

A number of preclinical findings have suggested the epidermal growth factor receptor (EGFR) as a target for drug development. Activation of EGFR promotes tumor cell growth, proliferation, and survival. Inhibition of EGFR results in tumor growth delay or regression. EGFR blockade potentiates the effect of chemotherapy or irradiation in cell culture and in animal models. In addition to being upregulated in breast and colorectal cancer, EGFR has been shown to be upregulated in 40-80 percent of NSCLC tumors. When bound by ligand, EGFR forms a homodimer or heterodimerizes with another EGFR family member, the kinase domain is activated, and tyrosine residues in the intracytoplasmic tail are phosphorylated. This leads to downstream signaling and a variety of changes characteristic of malignant progression including upregulation of ras, raf, mitogen-activated phosphorylated (MAP) kinase, and the

vascular endothelial growth factor. In turn, cellular growth, invasive capacity, and tumor angiogenesis are enhanced.

There are currently two clinically active inhibitors of EGFR tyrosine kinase activity, erlotinib and gefitinib. These drugs are thought to work by binding within the ATP cleft of the tyrosine kinase domain and specifically inhibiting EGFR tyrosine kinase activity. Two notable large scale trials of gefitinib activity are the Iressa Dose Evaluation in Advanced Lung Cancer (IDEAL)-1 and IDEAL-2 trials [1,5]. IDEAL-1 enrolled 210 patients in Europe and Japan and randomized the patients to 250 mg and 500 mg daily doses of gefitinib. The objective response rate in this trial was 18.4% for those receiving 250 mg and 19% for those receiving 500 mg. Of note, in Japanese patients, there was an increased response rate of approximately 27.5% as compared to 10.4% in Europeans. Similarly, IDEAL-2 evaluated the response to gefitinib in 216 patients in the United States. Patient responses were based on both standard radiographic criteria and their symptom improvement as measured by the Functional Assessment of Cancer Therapy – Lung (FACT-L) quality of life instrument. In the 250 mg and 500 mg groups symptomatic improvement was noted in 43% and 35% of patients respectively. The radiographic response rate (PR+CR) for the 250 mg group was 12% while the 500 mg group had a 9% response rate.

While the radiographic response rates were relatively low, responses were frequently dramatic and durable suggesting that a subset of patients who responded might have a peculiar molecular aberration resulting in sensitivity. In both IDEAL-1 and IDEAL-2, adenocarcinoma and female sex correlated with response. Retrospective analysis of patients at MSKCC who received gefitinib on clinical trials found that certain clinical characteristics predicted response [6]. These factors included being a never smoker and the presence of bronchioalveolar features within an adenocarcinoma.

Recently, analysis of the sequence of the EGFR from patients with NSCLC has shown that mutations which affect the ATP binding cleft of the kinase domain are found in patients who have responded to gefitinib but not in other patients. Lynch et al. examined the sequence of tumor EGFR in 16 patients (9 gefitinib responders and 7 non-responders) [2]. They found that eight of the nine gefitinib responders had mutations clustered around the ATP-binding pocket of the tyrosine kinase domain. Four of these eight mutations comprised small deletions which removed four amino acids (residues 747-750). The remaining four were the result of single base changes, resulting in amino acid substitutions at amino acid 858, 861, or 719, all residues within the kinase domain of EGFR. These mutations were not seen in matched normal tissues. In the gefitinib non-responders, there were no such mutations. These findings suggest that these specific changes in the EGFR tyrosine kinase domain are associated with clinical responsiveness to gefitinib. The role of these EGFR mutations in tumor biology has not been fully elucidated.

Paez et al. performed a more wide ranging EGFR sequence analysis of patients with NSCLC [3]. They sequenced the EGFR genes in unselected NSCLC patients, 58 tumors from Japan

and 61 from the United States. They found tumor-specific EGFR mutations in 15 of the 58 tumors from Japan and 1 of the 61 U.S. tumors. Again, these mutations were found in portions of the gene coding for the ATP binding pocket. They went on to analyze the sequence of five gefitinib responders finding that all five had EGFR kinase domain mutations. These mutations included deletions in amino acid 747 to 750 (four of the five) and a point mutation at leucine 858 (one of five), demonstrating a relatively homogeneous pool of mutations which confer gefitinib susceptibility. In four patients who did not respond, there were no such mutations. The increased frequency of these mutations in a sample of tumors from Japanese patients may explain the significantly higher response rate to gefitinib in Japanese patients that was seen in IDEAL-1.

Finally, in a pilot study performed here by (W. Pao and H. E. Varmus, unpublished data) the EGFR gene sequence was analyzed in paraffin-embedded tumor tissue derived retrospectively from patients who responded to EGFR-TKIs. In one of three individual tumors examined, an 18-basepair deletion was identified within the EGFR kinase domain. This patient had experienced a durable radiographic regression with gefitinib. This deletion was not present in the patient's peripheral blood DNA, suggesting that the mutation developed in somatic cells. This mutation leads to an in-frame deletion of residues critical for ATP-binding and is similar to the deletion described by both Paez et al. and Lynch et al.

Unfortunately, nearly all patients who initially respond to gefitinib will eventually have progression of disease. The mechanism of development of resistance to these tyrosine kinase inhibitors is not currently known and is the subject of this protocol. Recent studies regarding another tyrosine kinase inhibitor, imatinib, in the treatment of chronic myelogenous leukemia (CML) and gastrointestinal stromal tumors (GIST) may be analogous. Imatinib is a relatively specific inhibitor of BCR-ABL and KIT, molecules that have been shown to be critical to the pathogenesis of these diseases. While most patients treated in chronic phase achieve a comlete hematologic remission, most patients in blast crisis either fail to respond or quickly relapse following an initial response to imatinib [7].

In CML, a host of mechanisms of resistance to imatinib have been postulated. However, the most frequent clinical finding is a clonal expansion of leukemic cells with mutations in the BCR-ABL oncogene. These mutants of BCR-ABL have changes which modify binding of the drug to the kinase domain. Specifically, Gorre et al. reported six of nine patients with a single identical mutation in ABL nucleotide 944 which resulted in a change from threonine to isoleucine at amino acid 315 of c-ABL [8]. The three remaining patients in this study had amplification of the bcr-abl gene. Followup studies have demonstrated a relatively finite number of mutations in bcr-abl which confer imatinib resistance. Other possible mechanisms of resistance to imatinib include increased drug efflux, altered serum protein binding, and loss of dependence of the CML clone on BCR-ABL [9].

Understanding the mechanism of resistance to gefitinib and erlotinib will allow more rational decisions about therapy of NSCLC with tyrosine kinase inhibitors. As part of this protocol,

we will examine post-treatment specimens for changes in EGFR gene sequence or copy number to correlate with resistance to gefitinib or erlotinib. If there is no evidence of compensatory mutations in EGFR or increased EGFR gene copy number, resistance may be explained by other mechanisms including pharmacologic mechanisms or mutations in other molecules downstream from EGFR in the MAPK pathway. Understanding the mechanism of resistance may alter how we approach the patient with progression of disease on erlotinib or gefitinib.

This protocol is designed to test the hypothesis: Changes in EGFR sequence in critical exons are related to acquired clinical resistance to gefitinib and erlotinib.

4.0 OVERVIEW OF STUDY DESIGN/INTERVENTION

4.1 Design

This is a protocol to obtain tissue specimens to identify changes in tumor DNA in NSCLC patients who have previously responded to either gefitinib or erlotinib therapy and who have subsequently experienced disease progression. We estimate that approximately 10 patients treated with erlotinib and 10 patients treated with gefitinib will be required to identify a pattern of changes in the mutations in EGFR. All patients seen by the Thoracic Oncology Service who have received erlotinib or gefitinib and who have experienced objective regression by RECIST, WHO or clinical criteria and who then progress will be screened for this protocol [10]. All patients entered on this trial will have tissue previously obtained which will allow identification of baseline EGFR sequence. Patients treated with gefitinib will have consented to this analysis of baseline tissue using IRB # 92-55. Those patients treated with erlotinib will have consented as part of IRB # 02-010.

4.2 Intervention

Patients who meet inclusion criteria will be assessed for sites of disease that are amenable to biopsy. After obtaining informed consent, biopsy will be obtained in the usual manner.

RNA isolation will be carried out using approximately 25 mg of gross tissue from these tumor specimens. RNA extraction will performed using TRIZOL reagent (Gibco-BRL, Gaithersburg, MD) and the Qiagen RNAeasy Kit (Qiagen, Hilden, Germany). RNA purity and quality will be assessed by spectrophotometry at A260 and A280, and by electrophoresis on a denaturing polyacrylamide gel (Agilent Technologies, Palo Alto, CA). EGFR mutational analysis: mRNA will be isolated as outlined above. The individual exons of the EGFR gene will be amplified by RT-PCR and sequenced using standard methods. If sufficient nucleic acid is available, sequencing of the coding regions of the following genes will also be performed: HER2, HER3, HER4, and KRAS.

EGFR gene copy number will be analyzed by Dr. Marc Ladanyi's lab. Chromogenic *in situ* hybridization (CISH) for EGFR gene amplification will be performed on paraffin sections using commercial reagents (EGFR CISH kit, Zymed Laboratories). The EGFR CISH signals will be counted in at least 30 nuclei with a light microscope using a 40X objective. Tumors are interpreted as positive for EGFR amplification when the average number of gene copies is >5 per nucleus.

5.0 THERAPEUTIC/DIAGNOSTIC AGENTS

Not Applicable

6.0 CRITERIA FOR SUBJECT ELIGIBILITY

6.1 Subject Inclusion Criteria

Patients diagnosed with unresectable or metastatic non-small cell lung cancer (NSCLC) and who fulfill the following eligibility criteria will be considered eligible for this study.

- Previously received treatment with erlotinib or gefitinib (patients may have received other treatments since erlotinib or gefitinib including radiation or chemotherapy)
- Radiologic partial or complete response to treatment with erlotinib or gefitinib as defined by RECIST or WHO.
- Radiologic progression of disease while on treatment as defined by RECIST or WHO
- Signed informed consent

Radiologic response criteria are based on previously published guidelines [10].

6.2 Subject Exclusion Criteria

- Patients who are unable to consent to biopsy or for whom repeat biopsy would be medically unsafe will be excluded.

7.0 RECRUITMENT PLAN

Potential research subjects will be identified by a member of the patient's treatment team, the protocol investigator, or research team at Memorial Sloan-Kettering Cancer Center (MSKCC). If the investigator is a member of the treatment team, s/he will screen their patient's medical records for suitable research study participants and discuss the study and their potential for enrolling in the research study. Potential subjects contacted by their treating physician will be referred to the investigator/research staff of the study.

The principal investigator may also screen the medical records of patients with whom they do not have a treatment relationship for the limited purpose of identifying patients who would be eligible to enroll in the study and to record appropriate contact information in order to approach these patients regarding the possibility of enrolling in the study.

During the initial conversation between the investigator/research staff and the patient, the patient may be asked to provide certain health information that is necessary to the recruitment and enrollment process. The investigator/research staff may also review portions of their medical records at MSKCC in order to further assess eligibility. They will use the information provided by the patient and/or medical record to confirm that the patient is eligible and to contact the patient regarding study enrollment. If the patient turns out to be ineligible for the research study, the research staff will destroy all information collected on the patient during the initial conversation and medical records review, except for any information that must be maintained for screening log purposes.

In most cases, the initial contact with the prospective subject will be conducted either by the treatment team, investigator or the research staff working in consultation with the treatment team. The recruitment process outlined presents no more than minimal risk to the privacy of the patients who are screened and minimal PHI will be maintained as part of a screening log. For these reasons, we seek a (partial) limited waiver of authorization for the purposes of (1) reviewing medical records to identify potential research subjects and obtain information relevant to the enrollment process; (2) conversing with patients regarding possible enrollment; (3) handling of PHI contained within those records and provided by the potential subjects; and (4) maintaining information in a screening log of patients approached (if applicable).

8.0 PRETREATMENT EVALUATION

Not applicable

9.0 TREATMENT/INTERVENTION PLAN

Patients who meet inclusion criteria will be assessed for sites of disease that are amenable to biopsy. After obtaining informed consent, a core biopsy will be obtained in the usual manner. Biopsy material will be processed to obtain RNA for analysis of EGFR sequence. Sequencing of additional candidate genes and other studies will follow if sufficient material is available.

10.0 EVALUATION DURING TREATMENT/INTERVENTION

Not applicable

Title of Table (bold)										
Test Type	Time 1	Non-Billable Research Charges	Time 2	Non-Billable Research Charges	Time 3	Non-Billable Research Charges	Time 4	Non-Billable Research Charges	Time 5	Non-Billable Research Charges
Pre-admission testing and Biopsy		Yes								

11.0 TOXICITIES/SIDE EFFECTS

11.1 The risks of a CT-guided core biopsy of a lung mass include:

- Bleeding
- Infection
- Pneumothorax
- Oversedation from narcotics and sedatives used during the procedure

12.0 CRITERIA FOR THERAPEUTIC RESPONSE/OUTCOME ASSESSMENT

Not Applicable

13.0 CRITERIA FOR REMOVAL FROM STUDY

Not applicable

14.0 BIOSTATISTICS

Gefitinib and Erlotinib are two active inhibitors of EGFR tyrosine kinase activity which results in tumor regression or growth delay. They are thought to work by binding within the ATP cleft of the tyrosine kinase domain and specifically inhibiting EGFR tyrosine kinase activity. Lynch et al. examined the sequence of tumor EGFR in 16 patients (9 gefitinib responders and 7 non-responders). They found that eight of the nine gefitinib responders had mutations clustered around the ATP-binding pocket of the tyrosine kinase domain. These mutations were not seen in matched normal tissues. In the gefitinib non-responders, there were no such mutations. These findings suggest that these specific changes in the EGFR tyrosine kinase domain are associated with clinical responsiveness to gefitinib. It is hypothesized that erlotinib behaves in a similar fashion. Unfortunately, nearly all patients who initially respond to gefitinib or erlotinib will eventually have progression of disease. The mechanism of development of resistance to these tyrosine kinase inhibitors is not currently known and is the subject of this protocol. It is designed to test the hypothesis: Changes in EGFR sequence in critical exons underlie acquired clinical resistance to gefitinib and erlotinib. To test this hypothesis EGFR sequence from tumor tissue pre-treatment in patients who respond to gefitinib or erlotinib will be compared to the EGFR sequence in tumor tissue post development of resistance. The binary response will be a 1 if there is a change and 0 if there is none.

Sample size calculation

It is hypothesized that the resistance to EGFR tyrosine kinase inhibitors is primarily due to a change in the EGFR sequence. (This is in line with resistance to imatinib in CML

patients.) We want to test whether the proportion of patients with a change is 30% (uninteresting) or 70% (interesting). If we sequence 9 patients pre- and post-resistance and at least 5 show a change then we can reject that the rate of change is only 30% (type I and II error rates are 10% each). Given that erlotinib and gefitinib may induce different mechanisms of resistance, we will aim to enroll 10 patients from each treatment group (this will account for poor RNA/DNA quality in one specimen per group).

Due to the small sample size the analyses of the data from EGFR gene copy number, gene expression level, and EGFR-related pathways will be exploratory only.

.

15.0 SUBJECT REGISTRATION AND RANDOMIZATION PROCEDURES

15.1 Subject Registration

The following person(s) can obtain informed consent:

Mark G. Kris, Vincent A. Miller, Naiyer A. Rizvi, Lee M. Krug, Jorge E. Gomez, Herbert F. Oettgen, Christopher G. Azzoli, William Pao, Gregory J. Riely, Valerie Rusch, Manjit Bains, Robert Downey, Raja Flores, Bernard Park

Confirm with electronic medical record that the patient has received the Notice of Privacy Practice. This must be obtained before the eligibility confirmation and obtaining of the research informed consent.

Confirm eligibility as defined in the section entitled Criteria for Patient/Subject Eligibility.

Obtain written informed consent, by following procedures defined in section entitled Informed Consent Procedures.

All patients must be registered through the Department of Medicine's (CTO) registration system at Memorial Sloan-Kettering Cancer Center. The CTO registry is available Monday through Friday from 8:15am - 5:00pm EST at 123-2150 (646) 227-2150 from outside MSKCC). The last page of the signed consent form, the signature page of the Research Authorization, and a completed Eligibility Checklist must be faxed to the CTO registry at the time of registration. The fax number is 212-557-0786.

During the registration process registering individuals will be required to answer specific eligibility questions and provide the following information:

Registering Individual	[Last, First Name]
Notice of Privacy Status	[Yes, No, N/A]
Research Authorization	[Date]
MSKCC IRB Protocol#	

Attending of Record (if applicable) [Last, First Name]
Consenting Professional [Last, First Name]
Informed Consent Date
Patient's Full Name [Last, First Name]
Patient MRN

15.2 Randomization

Not applicable

16.0 DATA MANAGEMENT ISSUES

A Research Study Assistant (RSA) will be assigned to the study. The responsibilities of the RSA include project compliance, data collection, abstraction and entry, data reporting, regulatory monitoring, problem resolution and prioritization, and coordinate the activities of the protocol study team.

The data collected for this study will be entered into a secure database. Source documentation will be available to support the computerized patient record.

16.1 Quality Assurance

Weekly registration reports will be generated to monitor patient accruals and completeness of registration data. Routine data quality reports will be generated to assess missing data and inconsistencies. Accrual rates and extext and accuracy of evaluations and follow-up will be monitored periodically throughout the study period and potential problems will be brought to the attention of the study team for discussion and action

Random-sample data quality and protocol compliance audits will be conducted by the study team, at a minimum of two times per year, more frequently if indicated.

16.2 Data and Safety Monitoring

The Data and Safety Monitoring (DSM) Plans at MSKCC were approved by the National Cancer Institute (NCI) in September 2001. The plans address the new policies set forth by the NCI in the document entitled "Policy of the National Cancer Institute for Data and Safety Monitoring of Clinical Trials" which can be found at:http://cancertrials.nci.nih.gov/researchers/dsm/index.html. The DSM Plans at MSKCC were established and are monitored by the Office of Clinical

Research. The MSKCC Data and Safety Monitoring Plans can be found on the MSKCC Intranet at: *http://mskweb2.mskcc.org/irb/index.htm* There are several different mechanisms by which clinical trials are monitored for data, safety and quality. There are institutional processes in place for quality assurance (e.g., protocol monitoring, compliance and data verification audits, therapeutic response, and staff education on clinical research QA) and departmental procedures for quality control, plus there are two institutional committees that are responsible for monitoring the activities of our clinical trials programs. The committees: *Data and Safety Monitoring Committee (DSMC)* for Phase I and II clinical trials, and the *Data and Safety Monitoring Board (DSMB)* for Phase III clinical trials, report to the Center's Research Council and Institutional Review Board. During the protocol development and review process, each protocol will be assessed for its level of risk and degree of monitoring required. Every type of protocol (e.g., NIH sponsored, in-house sponsored, industrial sponsored, NCI cooperative group, etc.) will be addressed and the monitoring procedures will be established at the time of protocol activation.

17.0 PROTECTION OF HUMAN SUBJECTS

Risks: This trial will require biopsy of tumor tissue for research purposes. Since these analyses will be performed on tumor tissue and not germline DNA and the genes to be evaluated are not involved in known hereditary disease processes, we foresee no implications for establishing the molecular phenotype of these tumors to the patients or their relatives.

Benefits: We propose no therapeutic benefit for the patients who participate in this research. Molecular observations relating to drug sensitivity and resistance mechanisms may identify genes important to the therapeutic planning of future patients. Therefore, the proposed benefits are for future patients with this disease.

Possible toxicities/side effects: Side effects and toxicities will relate to the type of biopsy procedure performed.

Confidentiality: Patient's names or any other personally identifying information will not be used in reports or publications resulting from this study. The Food and Drug Administration, other authorized agencies (e.g. qualified monitors from MSKCC), and appropriate personnel may review data as required.

This study does not include children because the number of children with NSCLC is limited and because the majority of pediatric cancer patients are already accessed through a nationwide pediatric cancer research network. This statement is based on exclusion 4b

of the NIH Policy and Guidelines on the Inclusion of Children as Participants in Research Involving Human Subjects.

17.1 Privacy

It is the responsibility of the Research Staff to ensure that protocol patients have received the Center's Notice of Privacy Practices. If the subject has not already done so, MSK personnel must try to obtain acknowledgment before the patient participates in this study.

MSKCC's Privacy Office may allow the use and disclosure of protected health information pursuant to a completed and signed Research Authorization form. The use and disclosure of protected health information will be limited to the individuals described in the Research Authorization form. A Research Authorization form must be completed by the Principal Investigator and approved by the IRB and Privacy Board.

17.2 Serious Adverse Event (SAE) Reporting

Any SAE will be reported to the IRB as soon as possible but no later than 7 days. The IRB requires a memo **or** Clinical Research Database (CRDB) SAE report sent to the IRB Chairman (IRB Office M2102) containing the following:

1. The initials of the subjects, patient MRN #, protocol # and title
2. The date the event occurred
3. A description of the SAE
4. An explanation of how the SAE was handled
5. A description of the subject's condition
6. Indication if the subject remains on the study
7. Indication if the event is considered related to the treatment (drug, device, intervention)
8. Indication if an amendment will need to be made to the protocol and/or consent form as a result

All SAEs <u>must</u> be entered into the CRDB SAE form page.

18.0 INFORMED CONSENT PROCEDURES

All patients will be required to sign a statement of Informed Consent that meets the requirements of the Code of Federal Regulations 21 CFR 50.25 (Elements of Informed Consent) and the IRB of this center. Informed consent may be obtained only by investigators designated on the IRB approved protocol. After the purpose, methods, risks, and benefits of the study have been explained to the patient in a satisfactory manner, including a review of the Informed Consent document, three copies of the Informed Consent shall be signed by both patient and investigator. Patients will be told verbally, and also on the Informed Consent document, that a family member may be contacted at the end of the trial to determine their survival status. One copy will be

returned to the patient, one will be placed in the patient's medical record, and the third copy will be delivered to the MSKCC Clinical Trials Office.

18.1 Research Authorization

Procedures for obtaining Research Authorization: Before any protocol-specific procedures are carried out, investigators and/or designated staff will fully explain the details of the protocol, study procedures, and the aspects of patient privacy concerning research specific information. In addition to signing the IRB Informed Consent, all patients must sign the Research Authorization component of the informed consent form. The Research Authorization requires a separate set of signatures from the patient. The original signed documents will become part of the patient's medical record, and each patient will receive a copy of the signed documents.

19.0 REFERENCE(S)

1. Kris, M. G. et al. Efficacy of Gefitinib, an Inhibitor of the Epidermal Growth Factor Tyrosine Kinase, in Symptomatic Patients with Non-Small Cell Lung Cancer, JAMA 2003; 290(16): 2149-2158

2. Lynch, T. et al. Activating Mutations in the epidermal growth factor receptor underlying responsiveness of non-small-cell lung cancer to gefitinib, New England Journal of Medicine 2004; 350(21)

3. Paez, J.G. et al. EGFR mutations in lung cancer: Correlation with Clinical Response to Gefitinib Therapy, Science Express April 19, 2004.

4. Jemal, A., et al., *Cancer Statistics, 2003.* CA Cancer J Clin, 2003. 53(1): p. 5-26.

5. Fukuoka et al. Multi-Institutional Randomized Phase II Trial of Gefitinib for Previously Treated Patients with Advanced Non–Small-Cell Lung Cancer Journal of Clinical Oncology 2003, 21:2237-2246

6. Miller, V.A. et al. Bronchioloalveolar Pathologic Subtype and Smoking History Predict Sensitivity to Gefitinib in Advanced Non–Small-Cell Lung Cancer, Journal of Clinical Oncology 2004: 1103-1109.

7. Kantarjian H, Sawyers C, Hochhaus A, et al. Hematologic and cytogenetic responses to imatinib mesylate in chronic myelogenous leukemia. New England Journal of Medicine. 2002;346:645-652.

8. Gorre et al. Clinical Resistance to STI-571 Cancer Therapy Caused by BCR-ABL Gene Mutation or Amplification, Science 2001, 293: 876-890

9. Hingorani, S.R. et al., Targeting oncogene dependence and resistance, Cancer Cell, 2003, 3: 414-417.

10. Therasse, P., et al. New Guidelines to Evaluate the Response to Treatment in Solid Tumors, Journal of the National Cancer Institute 2000; 92:205–16

20.0 APPENDICES

None

PATIENT INFORMED CONSENT FOR CLINICAL RESEARCH

1.0 INTRODUCTION

We have asked you to participate in this study because you have a specific type of lung cancer, known as non-small cell lung cancer and have taken gefitinib (IRESSA) or erlotinib (TARCEVA). The doctors at Memorial Sloan-Kettering Cancer Center study cancer and attempt to develop improved treatments. This is called clinical research. To make an informed judgment about whether or not you want to be part of this study, you should understand its risks and benefits and the alternatives. This process is known as informed consent. This consent form gives detailed information about the research study. Once you understand the study, its risks, benefits and alternatives, you will be asked to sign this form if you wish to take part. You will be given a copy to keep.

The research study being proposed to you is:

Molecular Determinants of Acquired Clinical Resistance to Erlotinib and Gefitinib in Non-Small-Cell Lung Cancer

2.0 PURPOSE OF THE RESEARCH

Researchers at Memorial Sloan-Kettering Cancer Center are trying to learn more about how erlotinib and gefitinib work in treating lung cancer. Some early studies have shown that gefitinib is more likely to work if a particular DNA change (also known as a mutation) is found in a protein that is important in lung cancer. This protein is called the epidermal growth factor receptor (EGFR). Since gefitinib and erlotinib sometimes stop working, we would like to examine your tumor to learn why these medicines are not working as well. Your tumor will be examined for a variety of things including changes in the DNA of the EGFR. We will also sequence parts of the genes for HER2, HER3, HER4, and KRAS, other proteins thought to be important in lung cancer.

3.0 DESCRIPTION OF THE RESEARCH PROCEDURES

After you have given informed consent for this research protocol, a core needle biopsy of your lung cancer will be obtained. Prior to the biopsy, you will talk with the physician performing the biopsy. You will be given a chance to talk about the procedure and its side effects. A separate consent form will be signed once you have agreed to the procedure. The material from this biopsy will be taken to the laboratory and studied.

4.0 SIDE EFFECTS/RISKS/BENEFITS

At this time, there is no evidence that this biopsy will directly benefit you.

5.0 COMPENSATION IN CASE OF RESEARCH-RELATED INJURY

If you are injured because of any substance or procedure during your participation in this study, medical care will be provided to you. Your health insurance company will be charged for this care. You will not receive any injury compensation. You should also understand that this is not a waiver of your legal rights.

6.0 ALTERNATIVES

If you do not wish to take part in this trial, not participating in this trial is an alternative.

7.0 FINANCIAL COST

You will not be charged for the biopsy, anesthesia, or pathology costs as a part of this clinical trial. You will not be charged for the research tests being performed including gene analysis of EGFR, HER2, HER3, HER4, and KRAS. If you need hospitalization or any other medical care as a result of this biopsy, your health insurance company will be charged for this care.

8.0 CONFIDENTIALITY/PRIVACY

Participation in research may cause a loss of privacy, but information about the patients will be kept as confidential as possible. Within the limits of the law, every effort will be made to keep your study records private. Neither your name nor any personal information will be used in any report or publication resulting from this work. Trained staff at Memorial Sloan-Kettering Cancer Center may review your records if necessary.

8.1 PRIVACY

It is the responsibility of the research staff at Memorial Hospital to ensure that your records are managed in such a way to protect your privacy. Access to your medical information will be limited to those listed in the Research Authorization Form which is a part of the informed consent process.

9.0 RIGHT TO REFUSE OR WITHDRAW/NEW FINDINGS

You may choose not to be in the study and if you do choose to participate, your participation is voluntary. You may also refuse to participate, or change your mind at any time. If you do not want to enter the study or decide to withdraw from the study, your relationship with the study staff will not change, and you may do so without penalty or loss of benefits to which you are otherwise entitled. If you do not want us to continue using the data we have already collected about you, you must withdraw your permission in writing.

10.0 TERMINATION

Not Applicable

11.0 CONCLUSION

Memorial Sloan-Kettering Cancer Center's Institutional Review Board is legally responsible for making sure that research with patients is appropriate and that the patients' rights and welfare are protected. It has reviewed this research study.

The physician in charge of this study is **Dr. Vincent A. Miller**, telephone number (212)-639-7243. If you need more information about this study before you decide to participate, or at any other time, you may feel free to contact him. A non-physician whom you may call for information about the consent process, research patients' rights, or research related injury is Janice Levy, telephone number (212) 639-5804.

PATIENT INFORMED CONSENT FOR CLINICAL RESEARCH

Molecular Determinants of Acquired Clinical Resistance to Erlotinib and Gefitinib in Non-Small-Cell Lung Cancer

Purpose: The purpose of this research is to compare the RNA of the epidermal growth factor receptor gene to that which was seen prior to treatment with gefitinib or erlotinib. Additionally, other studies of the molecular biology of your tumor may be performed.

Statement of professional obtaining consent

I have fully explained this research study to the patient or guardian of patient _____________________. In my judgment and the patient's or guardian's, there was sufficient access to information, including risks and benefits to make an informed decision.

Date: ________ Physician's Signature: ____

Physician's Name: ______________________________________
(Print)

Patient's/subject (or guardian's) statement

I have read the description of the clinical research study or have had it translated into a language I understand. I have also talked it over with the doctor to my satisfaction. I understand that my/the patient's participation is voluntary. I know enough about the purpose, methods, risks, and benefits of the research study to judge that I want (the patient/subject) to take part in it.

Patient/Subject number: ________ Patient/Subject Signature: _______________________

Date: _______________________ Patient's/Subject's Name: _______________________
(Print)

Molecular Determinants of Acquired Clinical Resistance to Erlotinib and Gefitinib in Non-Small Cell Lung Cancer

RESEARCH AUTHORIZATION

Patient Name: ______________________________ Patient MRN :____________________

We understand that information about you and your health is personal. We are committed to protecting the privacy of your information. Because of this commitment, we must obtain approval from you before we can use your protected health information for research purposes. This form provides that authorization. This form also helps us make sure that you are informed of how this information will be used or disclosed in the future. Please read the information below carefully before signing this form.

USE AND DISCLOSURE COVERED BY THIS AUTHORIZATION

*A representative of Memorial Sloan-Kettering Cancer Center must answer these questions completely before providing this authorization form to you. **PLEASE DO NOT SIGN A BLANK FORM**. You or your personal representative should read the descriptions below before signing this form.*

Who will have access to and/or use your health information?

The following individuals and/or organization(s) may have access to use, disclose or receive some information about you. They may only share the information to the individuals/parties indicated on this list. This information must be shared with you, the research subject and/or your personal representative, as required by law.

- Every research site for this study, including Memorial Sloan-Kettering Cancer Center and the research support staff (for example, research study assistant) and medical staff at each location
- Every health care personnel who provides services to you in connection with this study
- Any laboratories, other individuals/organizations that analyze your health information in connection with this study as defined by protocol
- The following research sponsor: Memorial Sloan-Kettering Cancer Center
- The National Cancer Institute and/or the National Institute of Health
- The United States Food and Drug Administration and other regulatory agencies responsible for oversight
- The members and staff of the hospital's Institutional Review Board and Privacy Board
- Principal Investigator and Co-Principal Investigator(s): Vincent A. Miller and Mark G. Kris

- Members of the Research Team including the participating investigators, research assistants, clinical nurses, fellows/residents and clerical support staff.
- Members and staff of the hospital's Office of Clinical Research including the research management and support staff in the clinical departments
- Members of the Hospital's Data Safety Monitoring Board/Committee and Quality Assurance Committee

What information will be used or disclosed?

The boxes checked below should provide you with enough detail so that you can understand what information may be used or disclosed.

☑ Your entire research record
☑ Any part of your medical records held by the hospital

☑ HIV-related information. This includes any information indicating that you have had an HIV-related test, or have HIV infection, HIV-related illness or AIDS, or any information which could indicate that you have been potentially exposed to HIV. (New York State requires us to obtain special consent.)

☑ The following information:

- biopsy specimen

SPECIFIC UNDERSTANDINGS

By signing this form, you give permission for the sharing of your protected health information noted above. The purpose for the use and disclosure of your information, is to conduct the research study explained to you during the informed consent process. This form also ensures that the information relating to the research is available to everyone who may need it. Your protected health information may also be used for your research treatment, to collect payment for your treatment while on the study (when applicable), and to run the business operations of the hospital.

Once we have shared your information with the individuals and organizations listed on this form, they may be able to share your information again, if they are not subject to laws that protect your privacy.

It is your right to refuse to sign this authorization form. If you do not sign this form, you will not be able to participate in the research study. You will not receive the research treatment that was described to you. Your health care outside the study will not be affected. The payment for your health care or your health care benefits will not be affected.

If you sign this authorization form, you will have the right to withdraw it at any time. To withdraw the authorization will prohibit further use or disclosure of your health information. If the hospital has already used your health information approved by your authorization or needs the information to fulfill an obligation or analyze the data, the use or disclosure can not be stopped. This authorization form will not expire unless you withdraw it. If you want to withdraw this authorization, please write to Dr. Vincent A. Miller, Department of Medicine at the hospital.

You have a right to see and copy your health information described in this authorization form in accordance with the hospital's policies. You also have a right to receive a copy of this form after you have signed it.

Notice Concerning HIV-Related Information

If you are authorizing the release of HIV-related information, you should be aware that the individuals/organizations are prohibited from sharing any HIV-related information without your approval unless permitted to do so under federal or state law. You also have a right to request a list of people who may receive or use your HIV-related information without authorization. If you experience discrimination because of the release or disclosure of HIV-related information, you may contact the New York State Division of Human Rights at (212) 480-2493 or the New York City Commission of Human Rights at (212) 306-7450. These agencies are responsible for protecting your rights.

SIGNATURE

I have read this form and all of my questions have been answered. By signing below, I acknowledge that I have read and accept all of the information above.

____
Signature of Subject or Personal Representative

____
Print Name of Subject or Personal Representative

____
Date

____
Description of Personal Representative's Authority

CONTACT INFORMATION

The contact information of the subject or personal representative who signed this form should be filled in below.

Address:
____
____
____

Telephone:
____________________ (daytime)
____________________ (evening)

Email Address (optional):
____

THE SUBJECT OR HIS OR HER PERSONAL REPRESENTATIVE MUST BE PROVIDED WITH A COPY OF THIS FORM AFTER IT HAS BEEN SIGNED.
